# Lower water-soluble vitamins and higher homocysteine are associated with neurodegenerative diseases

**DOI:** 10.1038/s41598-025-03859-y

**Published:** 2025-05-29

**Authors:** Cuiping Zhang, Yao Hu, Xinyi Cao, Yuhang Deng, Yuting Wang, Ming Guan, Xiaoyan Wu, Haoqin Jiang

**Affiliations:** 1 Department of Laboratory Medicine, Huashan Hospital, Shanghai Medical College, Fudan University, Shanghai, 200040 China; 2 Department of Laboratory Medicine, Qingpu District Hospital of Traditional Chinese Medicine, Shanghai, 201700 China

**Keywords:** Water-soluble vitamins, One-carbon metabolism, Homocysteine, cognitive impairment, Neurodegenerative diseases, Neuroscience, Medical research

## Abstract

Evidence of the effects of water-soluble vitamins (e.g., B vitamins, vitamin C, and total folate) on cognitive function in patients with neurodegenerative diseases is mixed. Furthermore, the relationships among homocysteine (Hcy) metabolism, water-soluble vitamins, and cognitive impairment remain unclear. Therefore, we aimed to investigate the role of the levels of water-soluble vitamins [e.g., vitamins B1, B2, B3, B5, B6, 5-methyltetrahydrofolate (5mTHF), B12, and C and total folate] and Hcy in dementia progression in patients with neurodegenerative diseases. In this retrospective cohort study, we enrolled 280 healthy controls and 646 patients with a neurodegenerative disease. Patients were classified into a Parkinson’s disease (PD) group (*n* = 312), Alzheimer’s disease (AD) group (*n* = 219), or other dementia group (*n* = 115) according to pathological features. The other dementia group comprised 25 patients with frontotemporal dementia, 38 with Lewy body dementia, 34 with vascular dementia, and 18 with semantic dementia. Serum vitamins (i.e., B1, B2, B3, B5, VB6, 5mTHF, and C) were measured via liquid chromatography-mass spectrometry/mass spectrometry. Total Hcy, vitamin B12 and total folate levels were measured using commercial electrochemiluminescence immunoassays. The serum levels of vitamins B1, B2, B5, B6, 5mTHF, and C were lower in all patient groups than in the control group. The logistic regression results revealed that lower levels of serum vitamins B2, B6, 5mTHF, and B12 were associated with a higher risk of dementia in PD patients, and higher Hcy levels and lower serum vitamin B6 and 5mTHF levels were associated with a higher risk of AD-related cognitive impairment. In addition, the level of vitamins was positively correlated with neuropsychological assessment scores and negatively correlated with Hcy level and stage of dementia. The levels of several water-soluble vitamins are lower in dementia patients. Moreover, lower levels of water-soluble vitamins and higher levels of Hcy increased odds ratios for having neurodegenerative diseases or cognitive impairment. These findings suggest that estimating water-soluble vitamin levels in older adults may be valuable given that they may help improve cognitive function.

**Supplementary Information:**

The online version contains supplementary material available at 10.1038/s41598-025-03859-y.

## Introduction

Neurodegenerative diseases encompass a wide range of pathologically and clinically diverse diseases that are characterized by progressive neuronal loss and increased prevalence with age^1^. Although Alzheimer’s disease (AD) and Parkinson’s disease (PD) are the most prevalent neurodegenerative diseases, various others are also relatively common, such as frontotemporal dementia (FTD), progressive supranuclear palsy, dementia with Lewy bodies (DLB), vascular dementia (VaD), multiple system atrophy, corticobasal degeneration, and Huntington’s disease (HD)^2^. Evidence has shown that nutritional status significantly influences the risk of developing cognitive decline and dementia in older adults^3,4^. Many neurodegenerative conditions progress to cognitive decline and dementia, and the number of individuals with dementia is expected to rise to 150 million people globally by 2050, which will cause enormous societal and economic burdens^5^. Currently, there are no adequate treatments for cognitive decline. Thus, developing better preventative strategies, novel diagnoses, and therapies has become a priority^6^. Furthermore, identifying individuals at high risk of cognitive decline and preventing dementia progression are major future challenges.

B vitamins are essential coenzymes for carbohydrate and amino acid catabolism and gluconeogenesis^7^. Because B vitamins cannot be synthesized by mammals, its deficiency can result in various negative effects, such as nervous and cardiovascular system dysfunction^8,9^. Neurotropic B vitamins are crucial coenzymes within the nervous system. Specifically, vitamins B1, B6, and B12 contribute to maintaining brain function and are involved in numerous neurometabolic pathways^10^. Vitamin B1 deficiency decreases glucose metabolism in the brain and causes neurological deficits similar to those observed in AD^11^. Vitamins B6, 5mTHF, and B12 play important roles in one-carbon metabolism. Deficiencies in B vitamins and folate are correlated with high homocysteine (Hcy) levels, which are risk factors for cerebrovascular dysfunction and cognitive impairment^12,13^. Several studies have indicated that B vitamins are important in decreasing the risk of developing cognitive impairment and is associated with higher cognitive function in older adults^14,15^. However, findings of an association between B vitamins and alterations in cognitive function are inconsistent across studies. For example, a meta-analysis revealed no significant overall effect of B vitamins supplementation on cognitive function^16^, whereas another study found that B vitamins supplementation slows cognitive decline, especially in those who receive early intervention^17^. To date, few cohort studies on the overall effects of water-soluble vitamins on different neurodegenerative diseases have been conducted. Furthermore, the associations among Hcy metabolism, water-soluble vitamins, and cognitive impairment remain unclear. Therefore, more comprehensive studies are needed to clarify the complex relationship between water-soluble vitamins and neurological degenerative disorders.

To better understand the relationship between water-soluble vitamins (e.g., vitamins B1, B2, B3, B5, B6, 5mTHF, B12, and C and total folate) and neurodegenerative diseases, we performed a cohort study involving 280 healthy controls and 646 patients with a neurodegenerative disease. We explored the association between vitamin levels and the risk of cognitive impairment for different groups of neurodegenerative disorder patients. Additionally, we explored the correlation between vitamin levels and dementia characteristics, such as neuropsychological examination scores, disease severity, and dementia ratings. Thus, the present study aims to present the distributions of B vitamins levels and the related variables and evaluate their risk value of different types of neurodegenerative diseases.

## Materials and methods

### Participants and subjects

The participants were recruited from Huashan Hospital of Fudan University between 2021 and 2023. Healthy controls were recruited from the health management center. We enrolled 280 healthy controls and 646 patients with a neurodegenerative disease. Patients were classified into a PD group (*n* = 312), AD group (*n* = 219), or other dementia group (*n* = 115) according to clinical features. PD patients were divided into two groups according to their cognitive condition: PD without dementia (PND; *n* = 235) and PD with dementia (PDD; *n* = 77). AD patients were subdivided into four groups according to their neuropsychological assessment scores: mild cognitive impairment (MCI; *n* = 18), mild AD (*n* = 17), moderate AD (*n* = 94), and severe AD (*n* = 90). The other dementia group comprised 25 patients with frontotemporal dementia (FTD), 38 with Lewy body dementia (LBD), 34 with vascular dementia (VaD), and 18 with semantic dementia (SD). The criteria for enrollment were as follows: (1) the diagnosis of the neurodegenerative disease was made by a neurologist, psychiatrist, or psychologist according to international clinical diagnostic criteria^18,19^; (2) the subject had undergone neurological and neuropsychological evaluations, which included the Mini-Mental State Examination (MMSE), Montreal Cognitive Assessment (MoCA), Hoehn-Yahr (HY) classification, Unified Parkinson’s Disease Rating Scale Part III (UPDRS-III), Boston Naming Test (BNT), and Clinical Dementia Rating (CDR) scale; (3) cognitive impairment characterized by a gradual onset and progression over the past year, an MMSE score of ≤ 26, and impaired performance of daily activities. We excluded subjects who had brain infarcts, hematoma tumors, or other complications, such as stroke, and recent infection or surgery. The study was approved by the Ethics Committee of Huashan Hospital, Fudan University (KY2023-515). All participants provided informed consent for participation in the study. The study was conducted in accordance with the ethical standards for medical research involving human subjects, as laid out in the 1964 Declaration of Helsinki and its later amendments.

### Measurement of serum vitamins

The serum samples of participants were obtained from abandoned blood samples with in vitro clinical diagnosis. Serum vitamins including thiamine (VB1), riboflavin (VB2), nicotinamide (VB3), pantothenic acid (VB5), 4-pyridoxic acid (VB6), biotin (VB7), 5-methyltetrahydrofolate (5mTHF, a specific form of VB9) and ascorbic acid (VC) were measured using a water-soluble vitamin determination kit (Shandong Yingsheng Biotechnology Co., Ltd., Jinan, China) and a liquid chromatography-mass spectrometry/mass spectrometry (LC-MS/MS) system (Waters Technologies Corporation, Milford, MA, USA) according to the instructions. Briefly, internal standard solution (60µL) was added to aliquots (60µL) of calibrators, controls, and serum in 1.5mL tubes (Axygen, Corning, NY, USA), and vortexed for 5 min. Then, the samples were centrifuged at 12,000 g for 10 min at 4 °C. 70µL of the supernatant was obtained and transferred to 96-well plate, and analyzed by LC–MS/MS. In the LC-MS/MS assay, the vitamin B9 measuring results were the concentration of 5mTHF, a dominant circulating form of vitamin B9^20^. The list of analytes, corresponding compound names, chemical abstracts service (CAS) numbers, and multiple reaction monitoring (MRM) transitions was concluded in Supplementary materials.

### Measurement of serum Hcy, vitamin 12 and total folate

Serum Hcy concentrations were analyzed with hydrolase-based enzymatic cycling method by Hitachi 7600 automatic analyzer (Hitachi Co., Ltd, Tokyo, Japan)^21^. The vitamin B12 and total folate were measured with the Alinity i system (Abbott Laboratories, IL, USA), an automated immunoassay analyzer utilizes chemiluminescent microparticle immunoassay (CMIA) principle, by using anti-analyte coated paramagnetic microparticles and anti-analyte acridinium-labeled conjugates^22^. The total folate measured with above immunoassays were comprised with all forms of folate that can bind to the folate-binding protein^23,24^. In this retrospective study, healthy population did not receive the measurements of VB12 and total folate.

### Statistical analysis

Statistical analyses were performed using Statistical Package for the Social Sciences version 26.0 (IBM Corp., Armonk, NY, USA). We first performed an analysis of variance and normal distribution test. The data are presented as means ± standard deviations or medians (25th and 75th percentiles) depending on the homogeneity and normality of the variance. The differences between two groups were examined via the Mann‒Whitney *U* test or independent *t* test. To determine differences among three or more groups, we used the Kruskal‒Wallis test, followed by *post hoc* tests with Bonferroni multiple comparison correction. We used binary logistic regression to calculate odds ratios (ORs) and 95% confidence intervals (CIs) for the association between serum vitamins and Hcy concentrations and dementia risk. With the binary logistic regression model, the age and sex were used as covariates to control the bias^25^. The healthy controls or patients without dementia were used as the reference groups. For the statistical analysis, the binary categories were replaced with integers (0 or 1) for group (case or control) and sex (male or female). Spearman correlation was used to analyze the relationships among vitamin levels and biochemical indicators. Differences were considered statistically significant at *p < 0.05, **p < 0.01, and ***p < 0.001.

## Results

### Patients with neurodegenerative diseases show lower serum vitamins but increased Hcy levels

**Fig. 1:**
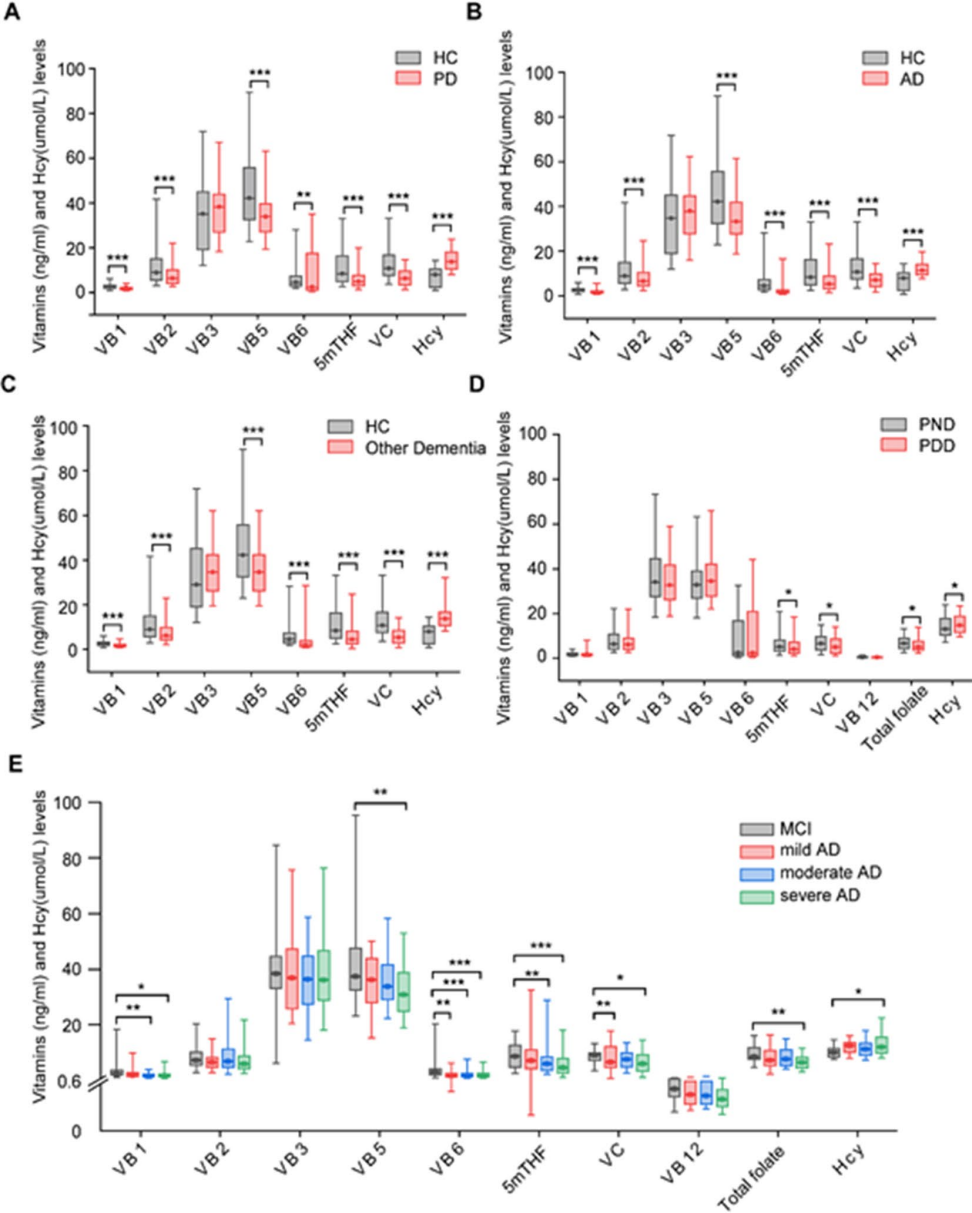
The levels of vitamins and Hcy in controls and neurodegenerative disease groups. (**A**) Serum vitamin and Hcy levels in patients with PD and controls. (**B**) Serum vitamin and Hcy levels in patients with AD and controls. (**C**) Serum vitamin and Hcy levels in patients with other forms of dementia and controls. (**D**) The levels of vitamins and Hcy in PND and PDD patients. (**E**) The levels of vitamins and Hcy in AD patients at different stages of cognitive impairment. *p < 0.05, **p < 0.01, ***p < 0.001. *HC* healthy controls, *VB1* vitamin B1, *VB2* vitamin B2, *VB3* vitamin B3, *VB5* vitamin B5, *VB6* vitamin B6, *5mTHF* 5-methyltetrahydrofolate, *VB12* vitamin B12, *VC* vitamin C, *Hcy* homocysteine.

We compared the serum vitamin levels between PD patients and controls. The clinical characteristics of the PD patients are presented in Table 1. The median age was higher in PD patients than in controls (65 years versus 60 years); however, there was no difference in sex. The levels of vitamins B1, B2, B5, B6, 5mTHF, and C were lower in the PD patients than in controls. The level of serum Hcy was significantly higher in the PD group than in the control group (Fig. 1A). Patients with AD were more likely to be women (*p* = 0.003). No difference was found between the AD and control groups in terms of age (Table 1). We also found that the levels of vitamins B1, B2, B5, B6, 5mTHF, and C were lower and that the serum Hcy level was higher in AD patients than in controls (Fig. 1B). The other dementia group characteristics are provided in Table 1. The levels of vitamins B1, B2, B5, B6, 5mTHF, and C were lower and the serum Hcy level was higher in patients with other forms of dementia than in healthy controls (Fig. 1C).

**Table 1:** Clinical characteristics and serum vitamin levels in patients with dementias and controls.

Variable	Control (*n* = 280)	PD (*n* = 312)			AD (*n* = 219)			Other dementia(*n* = 115)		
	M (P25, P75)	M (P25, P75)	χ2 or Z score	*p* value	M (P25, P75)	χ2 or Z score	*p* value	M (P25, P75)	χ2 or Z score	*p* value
Age (year)	60.00 (55.00, 66.75)	65.00 (57.00, 71.00)	− 3.739	**< 0.001**	59.00 (55.00, 68.00)	− 0.017	0.987	67.00 (59.00, 72.00)	− 5.443	**< 0.001**
Sex (n)	Male: 161	Male: 193	1.166	0.280	Male:97	0.585	**0.003**	Male: 75	2.019	0.155
	Female: 119	Female: 119			Female:122			Female: 40		
VB1 (ng/mL)	2.50 (2.00, 3.28)	1.70 (1.30, 2.20)	− 8.880	**< 0.001**	1.80 (1.30, 2.30)	− 6.988	**< 0.001**	1.70 (1.30, 2.30)	− 5.894	**< 0.001**
VB2 (ng/mL)	9.00 (5.61, 15.08)	6.40 (4.13, 10.08)	− 5.499	**< 0.001**	6.60 (4.50, 10.50)	− 4.733	**< 0.001**	6.20 (4.30, 9.80)	− 3.950	**< 0.001**
VB3 (ng/mL)	34.69 (19.13, 45.13)	38.10 (27.00, 44.15)	− 3.554	0.057	37.75 (27.90, 45.10)	− 4.061	0.070	32.20 (23.70, 41.40)	− 1.381	0.167
VB5 (ng/mL)	42.15 (32.50, 55.85)	33.90 (27.13, 39.75)	− 8.069	**< 0.001**	33.30 (27.70, 41.80)	− 6.906	**< 0.001**	34.60 (26.20, 42.30)	− 5.188	**< 0.001**
VB6 (ng/mL)	4.54 (2.95, 7.33)	2.52 (1.13, 17.45)	− 3.229	**0.001**	1.89 (1.34, 2.73)	− 12.435	**< 0.001**	2.13 (1.37, 4.96)	− 7.507	**< 0.001**
5mTHF (ng/mL)	8.45 (4.93, 16.28)	4.85 (3.00, 7.70)	− 8.132	**< 0.001**	5.40 (3.30, 9.00)	− 5.828	**< 0.001**	4.50 (2.50, 8.10)	− 6.467	**< 0.001**
VC (ng/mL)	10.80 (7.60, 16.68)	6.30 (3.43, 9.60)	− 10.887	**< 0.001**	7.20 (4.30, 9.80)	− 9.181	**< 0.001**	5.40 (3.00, 8.40)	− 9.435	**< 0.001**
Hcy (µmol/L)	8.02 (2.43, 10.40)	13.75 (10.52, 18.02)	− 14.521	**< 0.001**	11.47 (9.49, 14.09)	− 10.382	**< 0.001**	13.73 (10.72, 16.75)	− 10.334	**< 0.001**

*VB1* vitamin B1, *VB2* vitamin B2, *VB3* vitamin B3, *VB5* vitamin B5, *VB6* vitamin B6, *5mTHF* 5-methyltetrahydrofolate, *VC* vitamin C, *Hcy* homocysteine.

Significant values are in bold.

### Dementia patients show lower serum vitamins but increased Hcy levels

As shown in Table 2, the PDD group was older than the PND group. The MMSE and MoCA scores were lower and the HY stages and UPDRS-III scores were greater in the PDD patients than in the PND patients. Neuropsychological assessment scores indicated that PDD patients had cognitive impairment. The levels of 5mTHF, vitamin C, and total folate were significantly lower and serum Hcy was significantly higher in PDD patients than in PND patients (Fig. 1D).

**Table 2:** Clinical characteristics and serum vitamin levels in patients with PND and PDD.

Variable	PND (*n* = 235)	PDD (*n* = 77)	χ2 or Z score	*p* -value
Age (year)	64.00 (54, 69)	69.00 (63, 73)	− 4.242	**< 0.001**
Sex (n)	Male: 139	Male: 54	2.694	0.085
	Female: 96	Female: 23		
MMSE	28.00 (26.00, 29.00)	20.00 (18.00, 24.00)	− 7.286	**< 0.001**
MOCA	22.00 (19.00, 26.00)	15.50 (10.75, 19.00)	− 6.652	**< 0.001**
H-Y stage	2.50 (2.00, 3.00)	3.00 (3.00, 4.00)	− 3.351	**0.001**
Duration	72.00 (24.00, 120.00)	48.00 (36.00, 84.00)	− 1.250	0.211
UPDRS score	42.50 (26.00, 53.00)	56.00 (47.00, 74.00)	− 2.504	**0.012**
VB1 (ng/mL)	1.60 (1.30, 2.20)	1.80 (1.30, 2.10)	− 0.222	0.824
VB2 (ng/mL)	6.40 (4.20, 10.80)	6.10 (4.05, 9.10)	− 0.298	0.766
VB3 (ng/mL)	34.10 (27.60, 44.50)	32.70 (26.20, 42.65)	− 1.062	0.288
VB5 (ng/mL)	32.80 (27.00, 39.00)	34.50 (27.60, 42.15)	− 1.156	0.248
VB6 (ng/mL)	2.53 (1.16, 16.85)	2.45 (1.05, 20.87)	− 0.655	0.512
5mTHF (ng/mL)	5.10 (3.20, 8.10)	4.10 (2.30, 7.20)	− 2.063	**0.039**
VC (ng/mL)	6.60 (3.80, 9.70)	5.00 (2.40, 8.65)	− 2.585	**0.010**
VB12 (ng/mL)	0.46 (0.32, 0.68)	0.44 (0.30, 0.59)	− 1.119	0.263
Total folate (ng/mL)	6.70 (4.40, 9.05)	5.10 (3.75, 7.85)	− 2.051	**0.040**
Hcy (µmol/L)	13.11 (10.28, 17.78)	14.80 (11.62, 18.70)	− 2.223	**0.026**

*VB1* vitamin B1, *VB2* vitamin B2, *VB3* vitamin B3, *VB5* vitamin B5, *VB6* vitamin B6, *5mTHF* 5-methyltetrahydrofolate, *VB12* vitamin B12, *VC* vitamin C, *Hcy* homocysteine.

Significant values are in bold.

We found that clinical characteristics; sex; MMSE, MOCA, CDR, and BNT scores; and disease duration differed significantly among the four AD groups (Table 3). Moderate and severe AD was more common in women than in men. The levels of vitamins B1, B5, B6, 5mTHF, and C, total folate, and Hcy were significantly different among the four groups. The levels of vitamin B5, 5mTHF, vitamin C and total folate tend to decreased with development of AD (Table 3). Table 4 shows the *p*-values of the multiple comparisons between patients with MCI and those with mild, moderate, and severe AD. The scores of the cognitive function assessments, including the MMSE, MOCA, and BNT, decreased with the development of AD dementia. The levels of vitamins B1 and 5mTHF were lower in moderate AD patients and severe AD patients than in MCI patients. The levels of vitamins B5 and C and total folate were lower in severe AD patients than in MCI patients. The level of vitamin B6 was significantly lower in mild, moderate, and severe AD patients than in MCI patients, although this was not dependent on AD stage (Fig. 1E).

**Table 3:** Clinical characteristics and serum vitamin levels of patients at different AD stages.

Variable	MCI (*n* = 18)	Mild AD (*n* = 17)	Moderate AD (*n* = 94)	Severe AD (*n* = 90)	χ2 value	*p*-value
Age (years)	63.50 (54.00, 72.50)	63.65 ± 11.34	58.00 (54.00, 68.00)	59.00 (56.00, 67.00)	1.81	0.612
Sex (n)	Male: 11	Male: 10	Male:41	Male:35	13.13	**0.011**
	Female: 7	Female: 7	Female:53	Female:55		
MMSE	24.00 (24.00, 26.25)	22.00 ± 1.29	15.00 ± 2.83	5.00 (3.00, 6.00)	184.82	**< 0.001**
MOCA	19.00 (15.75, 23.00)	17.00 (15.00, 18.00)	9.00 (6.0, 12.00)	3.00 (1.00, 4.00)	142.18	**< 0.001**
CDR	1.00 (1.00, 1.50)	1.00 (0.5, 1.00)	1.00 (1.00, 2.00)	3.00 (2.00, 7.00)	34.83	**< 0.001**
BNT	21.44 ± 4.76	20.00 ± 5.21	15.47 ± 4.76	10.00 (7.25, 12.00)	55.87	**< 0.001**
Duration (m)	9.08 ± 26.85	10.17 ± 14.70	24.00 (12.00, 36.00)	36.00 (24.00, 48.00)	20.29	**< 0.001**
Education (y)	8.00 (5.50, 14.50)	11.50 (5.75, 12.75)	7.50 (6.0, 12.00)	9.00 (5.75, 9.00)	0.94	0.816
VB1 (ng/mL)	2.30 (1.98, 3.78)	1.80 (1.45, 2.80)	1.60 (1.30, 2.33)	1.70 (1.30, 2.20)	11.61	**0.009**
VB2 (ng/mL)	8.33 ± 4.54	7.21 ± 3.68	6.85 (4.65, 11.48)	5.90 (4.20, 8.98)	2.13	0.546
VB3 (ng/mL)	38.85 (32.98, 51.73)	40.40 (25.80, 51.25)	36.35 (27.40, 44.73)	36.10 (28.58, 46.60)	1.22	0.747
VB5 (ng/mL)	38.60 (32.30, 62.15)	36.20 (28.05, 44.80)	33.75 (28.95, 41.73)	30.80 (24.75, 39.00)	10.97	**0.012**
VB6 (ng/mL)	2.91 (1.97, 4.22)	1.60 (1.23, 2.48)	1.85 (1.41, 2.62)	1.69 (1.26, 2.77)	8.71	**0.033**
5mTHF (ng/mL)	8.95 ± 4.54	7.10 (4.10, 13.20)	6.00 (3.70, 8.43)	4.55 (2.60, 8.05)	12.15	**0.007**
VC (ng/mL)	8.69 ± 2.44	8.089 ± 5.19	7.60 (4.85, 9.95)	6.00 (3.28, 9.20)	10.66	**0.014**
VB12 (ng/mL)	0.54 (0.43, 0.68)	0.47 (0.35, 0.62)	0.45 (0.34, 0.64)	0.41 (0.31, 0.54)	7.68	0.053
Total folate (ng/mL)	9.64 ± 3.43	8.13 ± 4.17	7.65 (5.40, 11.00)	6.45 (4.53, 8.30)	17.02	**0.001**
Hcy (µmol/L)	10.11 ± 2.17	12.09 ± 2.44	11.28 (9.07, 13.49)	12.00 (9.92, 15.70)	3.72	**0.012**

*VB1* vitamin B1, *VB2* vitamin B2, *VB3* vitamin B3, *VB5* vitamin B5, *VB6* vitamin B6, *5mTHF* 5-methyltetrahydrofolate, *VB12* vitamin B12, *VC* vitamin C, *Hcy* homocysteine.

Significant values are in bold.

**Table 4:** Multiple comparisons between patients with MCI, mild AD, moderate AD, and severe AD.

Variable	MCI versus (*p* value)		
	Mild AD	Moderate AD	Severe AD
MMSE	0.003	**< 0.001**	**< 0.001**
MOCA	0.306	**< 0.001**	**< 0.001**
CDR	0.426	0.730	**< 0.001**
BNT	0.727	**< 0.001**	**< 0.001**
Duration	0.642	0.492	**0.037**
VB1	0.469	**0.003**	**0.014**
VB2	0.946	0.780	0.996
VB3	0.904	0.343	0.790
VB5	0.266	0.054	**0.002**
VB6	**0.001**	**< 0.001**	**< 0.001**
5mTHF	0.056	**0.002**	**< 0.001**
VC	**0.002**	0.136	**0.018**
VB12	0.857	1.000	0.273
Total folate	0.352	0.350	**0.002**
Hcy	0.392	0.355	**0.018**

*VB1* vitamin B1, *VB2* vitamin B2, *VB3* vitamin B3, *VB5* vitamin B5, *VB6* vitamin B6, *5mTHF* 5-methyltetrahydrofolate, *VB12* vitamin B12, *VC* vitamin C, *Hcy* homocysteine.

Significant values are in bold.

### Lower serum vitamins or elevated Hcy are associated increased odds ratios for having neurodegenerative disease or cognitive deficits

**Fig. 2:**
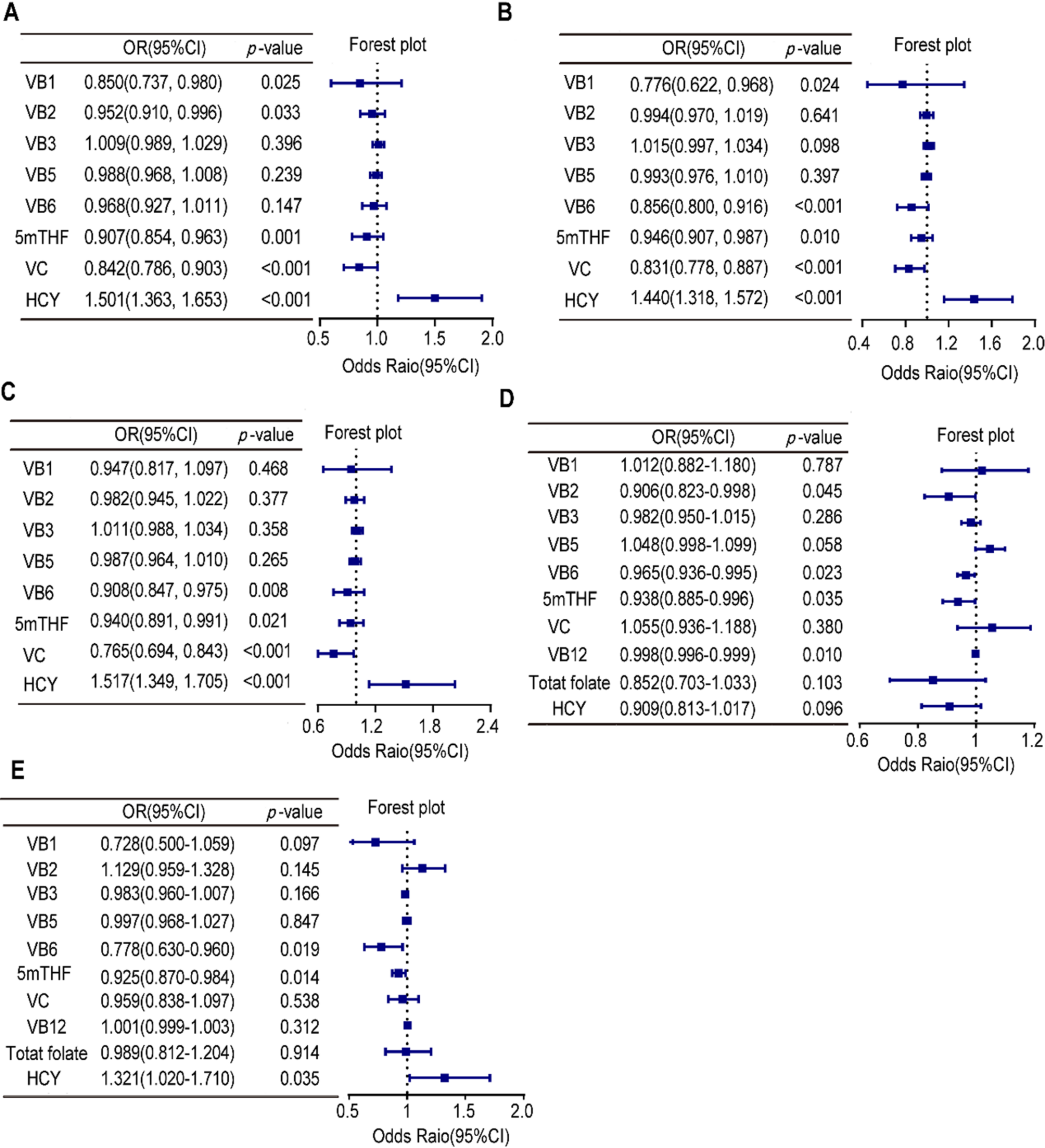
Forest plots showing ORs and 95% CIs for the risk of developing PD (**A**), AD (**B**), other forms of dementia (**C**), cognitive cognitive impairment in PD patients (**D**), and cognitive impairment in AD patients (**E**), based on the levels of vitamins and Hcy after adjusting for age and sex. *VB1* vitamin B1, *VB2* vitamin B2, *VB3* vitamin B3, *VB5* vitamin B5, *VB6* vitamin B6, *5mTHF* 5-methyltetrahydrofolate, *VB12* vitamin B12, *VC* vitamin C, *Hcy* homocysteine.

After adjusting for age and sex, the binary logistic regression model showed that lower levels of vitamins B1 (OR = 0.85, 95% CI 0.737–0.98, *p* = 0.025), B2 (OR = 0.952, 95% CI 0.91–0.996, *p* = 0.033), 5mTHF (OR = 0.907, 95% CI 0.854–0.963, *p* = 0.001), and C (OR = 0.842, 95% CI 0.786–0.903, *p* < 0.001) and a higher level of Hcy (OR = 1.501, 95% CI 1.363–1.653, *p* < 0.001) were associated with a greater risk of PD (Fig. 2A). Lower levels of vitamins B1 (OR = 0.776, 95% CI 0.622–0.968, *p* = 0.024), B6 (OR = 0.856, 95% CI 0.8–0.916, *p* < 0.001), 5mTHF (OR = 0.946, 95% CI 0.907–0.987, *p* = 0.01), and C (OR = 0.831, 95% CI 0.778–0.887, *p* < 0.001) were associated with a higher risk of AD, whereas a higher level of Hcy (OR = 1.44, 95% CI 1.318–1.572, *p* < 0.001) was associated with a higher risk of AD (Fig. 2B). Similarly, lower levels of vitamins B6, 5mTHF, and C and a higher level of Hcy were associated with a greater risk of dementia (Fig. 2C).

We subsequently investigated the association between vitamin levels and the risk of cognitive impairment in PD and AD patients. After adjusting for age and sex, the binary logistic regression model showed that, in PD patients, lower levels of vitamins B2 (OR = 0.906, 95% CI 0.823–0.998, *p* = 0.045), B6 (OR = 0.965, 95% CI 0.936–0.995, *p* = 0.023), 5mTHF (OR = 0.938, 95% CI 0.885–0.996, *p* = 0.035), and B12 (OR = 0.998, 95% CI 0.996–0.999, *p* = 0.01) were associated with the risk of developing dementia (Fig. 2D). When we classified the AD patients into early-stage (i.e., MCI and mild AD patients) and middle- and advanced-stage groups (i.e., moderate and severe AD patients), we found that lower levels of vitamins B6 (OR = 0.778, 95% CI 0.63–0.96, *p* = 0.019) and B9 (*OR* = 0.925, 95% *CI* 0.870–0.984, *p* = 0.014) and a higher level of Hcy (OR = 1.321, 95% CI 1.02–1.71, *p* = 0.035) were associated with a greater risk of AD-related cognitive impairment (Fig. 2E).

### Correlation analysis between vitamin levels and dementia characteristics

**Fig. 3:**
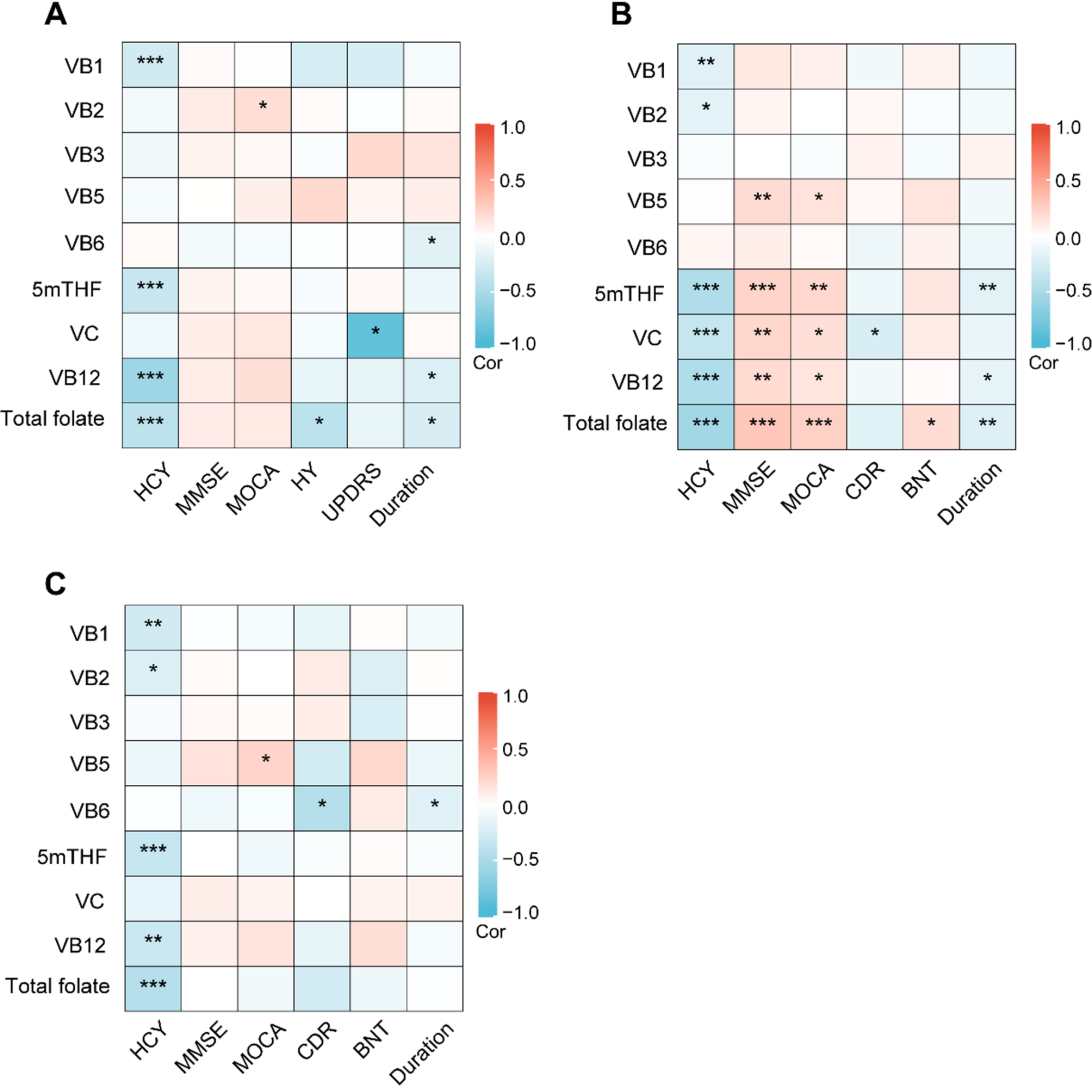
Heatmap for the Spearman correlation analysis between vitamin levels and dementia characteristics in PD patients (**A**), AD patients (**B**), and other dementia patients (**C**). *Hcy* homocysteine, *MMSE* Mini-mental State Examination, *MoCA* Montreal Cognitive Assessment, *HY* Hoehn-Yahr, *UPDRS* Unified Parkinson’s Disease Rating Scale Part III, *CDR* Clinical Dementia Rating, *BNT* Boston naming test, *VB1* vitamin B1, *VB2* vitamin B2, *VB3* vitamin B3, *VB5* vitamin B5, *VB6* vitamin B6, *5mTHF* 5-methyltetrahydrofolate, *VB12* vitamin B12, *VC* vitamin C. *p < 0.05, **p < 0.01, ***p < 0.001.

In PD patients, we found significant negative correlations between serum vitamin B1, 5mTHF, and B12 and total folate levels and the Hcy level. The level of vitamin B2 was positively correlated with the MoCA score. The level of total folate was negatively correlated with the HY stage. Serum vitamin C level was significantly negatively correlated with the UPDRS score. The levels of serum vitamins B6 and B12 and total folate were negatively correlated with disease duration (Fig. 3A and Supplementary Table 1). In addition, we observed noteworthy correlations between vitamin levels and AD-related cognitive function (Fig. 3B and Supplementary Table 2). In AD patients, the levels of vitamins B1, B2, 5mTHF, C, and B12 and total folate were negatively correlated with the Hcy level. The levels of vitamins B5, 5mTHF, C, and B12 and total folate were positively correlated with the MMSE and MoCA scores. Additionally, the level of vitamin C was negatively correlated with the CDR scale score. The level of total folate was positively correlated with the BNT score. The levels of vitamins 5mTHF and B12 and total folate were negatively correlated with disease duration. Similar results were obtained in patients with other dementias (Fig. 3C and Supplementary Table 3). The levels of vitamins B1, B2, 5mTHF, and B12 and total folate were negatively correlated with the Hcy level. The vitamin B5 level was positively correlated with the MoCA score. The level of vitamin B6 was negatively correlated with the CDR scale score and disease duration.

## Discussion

The aim of this retrospective study was to investigate the relationship between the levels of water-soluble vitamins and neurodegenerative diseases. We revealed that the levels of vitamins B1, B2, B5, B6, 5mTHF, and C in patients with PD, AD, and other neurodegenerative diseases, such as FTD, LBD, VaD, and SD, were lower than those in the control group. Our logistic regression results revealed that lower levels of vitamins B1, B2, B6, 5mTHF, and C and a higher level of Hcy were associated with a greater risk of developing a neurodegenerative disease. Specifically, in PD and AD patients, lower vitamin B6 and 5mTHF levels with a higher Hcy level were associated with a heightened risk of developing cognitive impairment. Furthermore, we found that low water-soluble vitamin levels were associated with poorer neuropsychological assessment scores, including the MMSE, MoCA, and BNT scores. Additionally, lower levels of water-soluble vitamins were associated with greater progression of cognitive decline, as measured by the HY staging, UPDRS score, CDR scale score, and disease duration.

The key risk factors for neurodegenerative diseases are related to age, sex, and blood Hcy levels^12,26^. Our results revealed that the age, sex, and Hcy level of patients with neurodegenerative diseases differed significantly from those of controls. Compared with the control group, patients with PD and those with other dementias were older. Aging-related neurodegeneration can cause motor and nonmotor impairments in patients with PD. Additionally, aging-induced alterations in mitochondrial dysfunction and microglial inflammation play significant roles in disease development^27,28^. In our cohort, the AD patients were more likely to be women, which is consistent with a higher frequency of AD in women than in men^29,30^. Although the mechanism underlying such variation is currently unclear, the differences in brain atrophy rates and steroid regulation may involve in the sexual bias of AD^31,32^. MCI was associated with an increased risk for AD dementia development^33^. Indeed, men and women with AD exhibit different psychiatric and cognitive symptoms; women show faster cognitive decline after a diagnosis of MCI or AD dementia^31^. We also observed similar trends in dementia development between men and women. The frequencies of moderate AD and severe AD were higher in women than in men. A high level of Hcy is a well-established cardiovascular risk factor and oxidative stress promoter and is associated with the incidence of cognitive impairment and dementia^34^. A recent study in AD patients confirmed that a high level of Hcy is significantly associated with dementia development^35^. In our study, the level of serum Hcy was significantly higher in the PD, AD, and other dementia groups than in the healthy controls, and this was particularly pronounced in the PD patients with dementia and the advanced-stage AD patients. We also found that patients with a higher level of Hcy had a greater risk of developing AD dementia, which is consistent with previous findings^35^. B vitamins deficiency is related to increased Hcy, which interferes with the blood‒brain barrier and increases the brain lesion load^36–38^. In line with this, our results revealed that the levels of vitamins B1, 5mTHF, and B12 and total folate were negatively correlated with the Hcy level in PD, AD, and other dementia disease groups.

**Fig. 4:**
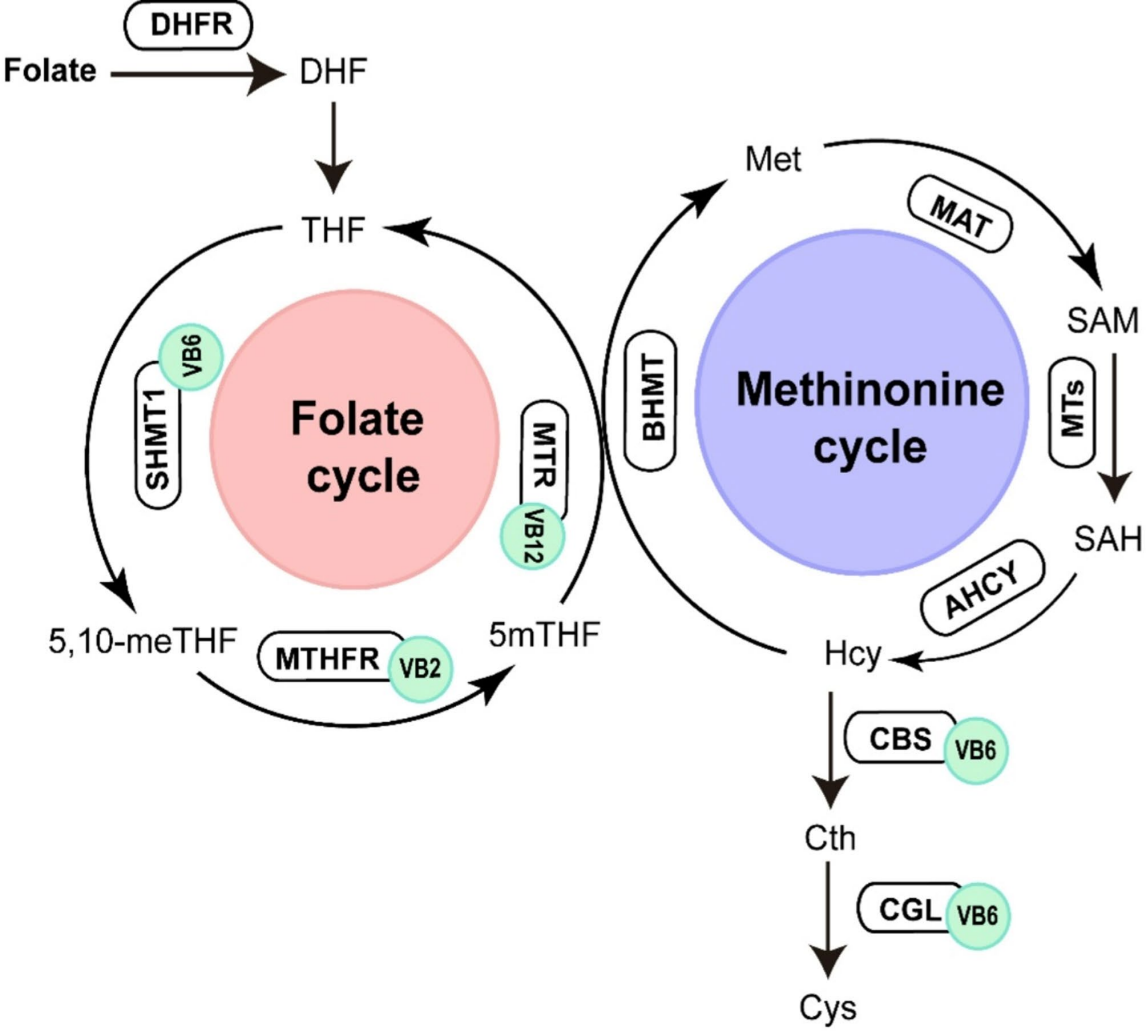
Schematic of the one-carbon metabolism pathways. The red circle represents the folate cycle. The blue circle represents the methionine cycle. The light green circles represent the coenzyme vitamins B2, B6, and B12. The black circles represent the enzymes involved in one-carbon metabolism. *DHFR* dihydrofolate reductase, *DHF* dihydrofolate, *THF* tetrahydrofolate, *SHMT1* serine hydroxymethyltransferase 1, *5*,*10-meTHF* 5,10-methylenetetrahydrofolate, *MTHFR* 5,10-methylenetetrahydrofolate reductase, *5mTHF* 5 methyl- tetrahydrofolate, *MTR* methionine synthase, *BHMT* betaine-homocysteine S-methyltransferase, *Met* methionine, *MAT* methionine adenosyltransferase, *SAM* S-adenosylmethionine, *MTs* methyltransferases, *SAH* S-adenosylhomocysteine, *AHCY* S-adenosyl-l-homocysteine hydrolase, *Hcy* homocysteine, *CBS* cystathionine β-synthase, *Cth* cystathionine, *CGL* cystathionine γ-lyase, *Cys* cysteine, *VB2* vitamin B2, *VB6* vitamin B6, *VB12* vitamin B12.

Vitamins B6 and B12 and total folate are involved in the one-carbon metabolic pathway (Fig. 4). One-carbon metabolism contributes significantly to cellular functions through the folate and methionine cycles^39^. Tetrahydrofolate (THF) converted into 5,10-methylenetetrahydrofolate (5,10-meTHF) with the help of vitamin B6-dependent enzyme, serine hydroxymethyltransferase 1 (SHMT1). Subsequently, 5,10-meTHF is transformed into 5mTHF by vitamin B2-dependent enzyme, 5,10-methylenetetrahydrofolate reductase (MTHFR) in the human body. The folate cycle is completed by the demethylation of 5mTHF by the vitamin B12-dependent enzyme methionine synthase (MTR). The demethylation process of 5mTHF provides the methyl donor for the remethylation of Hcy to methionine in the methionine cycle. Hcy can be converted to cystathionine (Cth) by cystathionine beta-synthase (CBS), which requires vitamin B6 as a cofactor. Cth is then converted to cysteine (Cys) with the enzyme cystathionine γ-lyase (CGL), which also requires vitamin B6. Therefore, deficiencies in vitamin B can disrupt the function of enzymes and increase the level of Hcy.

Previous studies have shown that plasma total folate and vitamin B12 levels are lower in patients with neurodegenerative diseases^40–42^. Our study revealed lower levels of vitamins B6 and 5mTHF and total folate in PD, AD, and other dementia patients. Moreover, insufficient serum vitamin 5mTHF and total folate levels in PD patients with dementia and reduced serum vitamin B6 and 5mTHF and total folate levels in AD patients are associated with cognitive decline. The level of vitamin B12 was lower in AD patients with advanced disease progression, although this difference was not significant (possibly because of the small sample size). There are several possible contributors to the deficiencies in one-carbon pathway-related vitamins in patients with cognitive impairment. Patients may experience reduced absorption of vitamin B12 and total folate because of gastrointestinal dysfunction, accompanied by changes in the gut microbiota^43,44^. The treatment of PD patients with levodopa may consume S-adenosylmethionine (SAM) as the methyl group donor in the methionine cycle, which consequently leads to Hcy accumulation^45,46^. Besides, the anti-PD drug, carbidopa irreversibly binds to and permanently deactivates Vitamin B6 and B6-dependent enzymes, leading to lower B6 concentrations^47^. Our results confirmed that lower levels of vitamins B6, 5mTHF, and B12 in PD patients and lower levels of vitamins B6 and 5mTHF and higher levels of Hcy in AD patients were associated with the risk of cognitive impairment and dementia progression. A previous study revealed that older individuals with lower concentrations of plasma vitamin B12 and folate perform poorly on cognitive assessments^48^. Deficiencies in vitamin B12 and folate cause an increase in the Hcy level because they play crucial roles in the remethylation pathway of Hcy metabolism. Hcy metabolism dysfunctions affect the aggregation of α-synuclein (αSyn), a protein that participates in dopaminergic neuron damage in PD and DLB^2,49–51^. In terms of AD pathophysiology, vitamin B12 and total folate reduce the burden of amyloid beta (Aβ) and tau protein-related neurofibrillary tangle deposition by alleviating mitochondrial oxidative stress^52,53^.

In our study, the measuring of total folate comprised with all forms of folate that can bind to the folate-binding protein based on the immunoassays, which is the most common method in clinical laboratories at present, because of its lower cost and laboratory technical requirement, rapid and automatic analysis procedures. However, the immunoassays have different binding affinity for different folate metabolites, which may reduce the specificity and lead to the deviation of the detection results^54^. In addition, the linear range of immunoassays is narrow, and the matrix effect may occur during sample dilution. LC-MS/MS can discriminate a single folic acid metabolite and eliminate the interference of structural analogues. The LC-MS/MS method has the advantages of high selectivity and detection specificity, good sensitivity and precision, so as to considered as the candidate reference method of folate metabolites detection^55^. Pervious study has investigated the comparability, precision, and accuracy of LC-MS/MS method in serum folate between different laboratories. They found that the measurement of 5mTHF shown better agreement and precision, less variable spiking recovery, and less bias by using a reference material^56^. Similar to pervious study, the results of total folate detection with immunoassays were agreement with results of 5mTHF detection with LC-MS/MS for the most parts^57^. Our study found that the level of serum 5mTHF was significantly decreased in moderate and severe AD, but the level of total folate was only significantly decreased in severe AD, when compared with MCI patients. The difference between the results of 5mTHF and total folate may due to different measurement methods. Our results indicated that detection of 5mTHF with LC-MS/MS to monitor serum folate level in AD patients may have higher sensitivity and specificity.

In neurodegenerative diseases, oxidative stress is one factor involved in various pathological changes, such as αSyn and Aβ deposition, inflammatory cytokine production, and blood‒brain barrier disruption^58–60^. The brain is susceptible to oxidative damage, and oxidative stress has been shown to facilitate the pathogenesis of dementia^61^. This has led to neuroprotective molecules with antioxidant and anti-inflammatory properties receiving increasing attention as preventive interventions for neurodegenerative diseases. Several vitamins, such as vitamins B1, B2, B3, B5, and C combat antioxidative effects and promote immune homeostasis^62–65^. Vitamin B1 deficiency is related to synaptic dysfunction and reduced choline acetyltransferase activity and neurogenesis, which involve both inflammation and oxidative stress in the brain and contribute to AD pathogenesis^11,66^. Therefore, vitamin B1 supplementation is beneficial for preventing inflammation and pathological conditions caused by oxidative stress^62^. Vitamin B2 is thought to protect against lipid oxidative stress and increase the glutathione level in brain tissue, which can improve cognitive performance^63^. Vitamin B3 is critical for myelination and dendritic development, cellular calcium signaling, DNA synthesis and repair, and strong antioxidant capacity in brain mitochondria^64,67^. Vitamin B5 (i.e., pantothenic acid) is an essential antioxidant vitamin that acts as the precursor of coenzyme A, which is a pivotal component of numerous metabolic pathways, such as the energy production and amino acid metabolism pathways^68^. Vitamin B5 deficiency in the brain has been observed in patients with neurodegenerative disorders involving myelin loss, such as AD, LDB, PDD, and HD^69–71^. Vitamin C is an essential nutrient for brain function, particularly because of its antioxidant mechanism. It is critical for neurodevelopment, neurotransmitter regulation, glutamate-mediated neurotransmission, and oxidative balance^72^. A meta-analysis showed that the level of vitamin C in patients with AD is significantly decreased^73^. Furthermore, a prospective study reported that a higher level of vitamin C is associated with a reduced risk of cognitive decline^74^. Similarly, our study revealed that the concentrations of vitamins B1, B2, and C were significantly lower in patients with PD, AD, and other forms of dementia than in the control group. We also observed a lower level of vitamin B3 in patients, although this did not reach significance. The level of vitamin C was particularly low in patients with PDD. Moreover, in AD patients, the lower the levels of vitamins B1, B5, and C, the greater the disease severity. Finally, the positive correlation between MMSE and MoCA scores and the levels of vitamins B2, B5, and C, and the negative correlation between the UPDRS and CDR scale scores and the vitamin C level suggest that a decrease in antioxidative vitamins is associated with cognitive decline.

This study has several limitations. Because this was a retrospective study, we are unable to determine from the data whether the changes in vitamin levels are a cause or a result of cognitive impairment in patients with neurodegenerative diseases. Importantly, vitamin levels can be affected by diet, season, and lifestyle. Obtaining cerebrospinal fluid and serum samples from the same participant would be necessary to determine the causality of alterations in the cerebral and peripheral systems. Another limitation is the lack of additional patient data, such as αSyn and Aβ condition, genetic background, and other factors that could have influenced the observations. In addition, neuropsychological assessment scores, such as MMSE and MoCA scores, and vitamin B12 and total folate measurements were not available for the control group. Future studies using a larger cohort to examine the relationship between vitamin levels and cognitive impairment are warranted.

## Conclusions

Our results suggest that the levels of several B vitamins are decreased in dementia patients and that lower levels of B vitamins and higher levels of Hcy increased odds ratios for having neurodegenerative disease or cognitive impairment. These findings underscore the importance of evaluating the level of B vitamins in older adults, especially given that they may help improve cognitive function. These findings provide support that B vitamins supplementation may be beneficial for preventing cognitive impairment in individuals with a neurodegenerative disease.

## Electronic supplementary material

Below is the link to the electronic supplementary material.

Supplementary Material 1

Supplementary Material 2

## Data Availability

All data supporting the findings of this study are available within the paper and its Supplementary Information.

## Abbreviations

Hcy: Homocysteine
PD: Parkinson’s disease
AD: Alzheimer’s disease
FTD: Frontotemporal dementia
LBD: Lewy body dementia
VaD: Vascular dementia
SD: Semantic dementia
HD: Huntington’s disease
MMSE: Mini-Mental State Examination
MoCA: Montreal Cognitive Assessment
HY: Hoehn-Yahr
UPDRS: Unified Parkinson’s Disease Rating Scale Part III score
BNT: Boston Naming Test
CDR: Clinical Dementia Rating
OR: Odds ratios
Cis: Confidence intervals
MCI: Mild cognitive impairment
DHFR: Dihydrofolate reductase
DHF: Dihydrofolate
THF: Tetrahydrofolate
SHMT1: Serine hydroxymethyltransferase 1
5,10-meTHF: 5,10-methylenetetrahydrofolate
MTHFR: 5,10-methylenetetrahydrofolate reductase
5mTHF: 5 methyl- tetrahydrofolate
MTR: Methionine synthase
BHMT: Betaine-homocysteine S-methyltransferase
Met: Methionine
MAT: Methionine adenosyltransferase
SAM: S-adenosylmethionine
MTs: Methyltransferases
SAH: S-adenosylhomocysteine
AHCY: S-adenosyl-L-homocysteine hydrolase
CBS: Cystathionine β-synthase
CGL: Cystathionine γ-lyase
Cys: Cysteine
HC: Healthy controls
