# Supplementary material for: Lower water-soluble vitamins and higher homocysteine are associated with neurodegenerative diseases
Source: Sci Rep. 2025 May 29;15:18866. doi: 10.1038/s41598-025-03859-y (PMC12122920; doi:10.1038/s41598-025-03859-y)
Supplement: Supplementary file 1 — Supplementary Material 1 [file 41598_2025_3859_MOESM1_ESM.docx]

Supplementary table 1. Correlation analysis between vitamins levels and dementia characteristics in PD patients

|  | **Hcy** | | **MMSE** | | **MOCA** | | **HY** | | **UPDRS** | | **Duration** | |
| --- | --- | --- | --- | --- | --- | --- | --- | --- | --- | --- | --- | --- |
|  | r | *p* | r | *p* | r | *p* | r | *p* | r | *p* | r | *p* |
| **VB1** | -0.272 | **<0.001** | 0.025 | 0.780 | 0.004 | 0.962 | -0.245 | 0.077 | -0.241 | 0.152 | -0.058 | 0.485 |
| **VB2** | -0.066 | 0.280 | 0.110 | 0.214 | 0.181 | **0.040** | 0.022 | 0.874 | -0.023 | 0.894 | 0.030 | 0.723 |
| **VB3** | -0.079 | 0.197 | 0.066 | 0.459 | 0.041 | 0.646 | -0.029 | 0.838 | 0.204 | 0.226 | 0.148 | 0.075 |
| **VB5** | -0.059 | 0.338 | 0.003 | 0.976 | 0.089 | 0.317 | 0.202 | 0.147 | 0.057 | 0.737 | 0.095 | 0.255 |
| **VB6** | 0.034 | 0.575 | -0.075 | 0.394 | -0.064 | 0.473 | -0.019 | 0.891 | -0.009 | 0.957 | -0.185 | **0.025** |
| **VB7** | -0.045 | 0.464 | -0.091 | 0.304 | -0.080 | 0.365 | 0.237 | 0.414 | -0.009 | 0.956 | 0.044 | 0.598 |
| **5mTHF** | -0.338 | **<0.001** | 0.068 | 0.444 | 0.040 | 0.654 | -0.048 | 0.731 | 0.037 | 0.830 | -0.104 | 0.211 |
| **VC** | -0.101 | 0.101 | 0.093 | 0.291 | 0.123 | 0.163 | -0.056 | 0.692 | -0.857 | **0.014** | 0.030 | 0.720 |
| **VB12** | -0.562 | **<0.001** | 0.098 | 0.322 | 0.167 | 0.089 | -0.142 | 0.446 | -0.146 | 0.539 | -0.215 | **0.019** |
| **Total folate** | -0.405 | **<0.001** | 0.101 | 0.306 | 0.115 | 0.245 | -0.406 | **0.023** | -0.137 | 0.564 | -0.235 | **0.010** |

Supplementary table 2. Correlation analysis between vitamins levels and dementia characteristics in AD patients

|  | **Hcy** | | **MMSE** | | **MOCA** | | **CDR** | | **BNT** | | **Duration** | |
| --- | --- | --- | --- | --- | --- | --- | --- | --- | --- | --- | --- | --- |
|  | r | *p* | r | *p* | r | *p* | r | *p* | r | *p* | r | *p* |
| **VB1** | -0.197 | **0.007** | 0.122 | 0.071 | 0.079 | 0.249 | -0.079 | 0.432 | 0.066 | 0.419 | -0.095 | 0.161 |
| **VB2** | -0.168 | **0.023** | 0.053 | 0.432 | 0.002 | 0.977 | 0.038 | 0.706 | -0.030 | 0.718 | -0.069 | 0.306 |
| **VB3** | -0.030 | 0.690 | <0.001 | 0.994 | -0.034 | 0.621 | 0.070 | 0.489 | -0.061 | 0.455 | 0.066 | 0.331 |
| **VB5** | 0.005 | 0.945 | 0.192 | **0.004** | 0.152 | **0.027** | 0.037 | 0.714 | 0.142 | 0.083 | -0.080 | 0.240 |
| **VB6** | 0.056 | 0.451 | 0.090 | 0.183 | 0.035 | 0.614 | -0.107 | 0.288 | 0.076 | 0.354 | -0.115 | 0.091 |
| **VB7** | -0.158 | **0.033** | 0.068 | 0.320 | 0.003 | 0.961 | -0.042 | 0.677 | 0.194 | **0.017** | -0.025 | 0.716 |
| **VB9** | -0.465 | **<0.001** | 0.228 | **0.001** | 0.211 | **0.002** | -0.102 | 0.311 | 0.129 | 0.115 | -0.179 | **0.008** |
| **VC** | -0.351 | **<0.001** | 0.218 | **0.001** | 0.175 | **0.011** | -0.235 | **0.019** | 0.106 | 0.198 | -0.126 | 0.062 |
| **VB12** | -0.471 | **<0.001** | 0.199 | **0.005** | 0.143 | **0.049** | -0.088 | 0.405 | 0.027 | 0.759 | -0.153 | **0.032** |
| **FA** | -0.548 | **<0.001** | 0.302 | **<0.001** | 0.249 | **0.001** | -0.180 | 0.087 | 0.183 | **0.035** | -0.200 | **0.005** |

Supplementary table 3. Correlation analysis between vitamins levels and dementia characteristics in other dementia diseases

|  | **Hcy** | | **MMSE** | | **MOCA** | | **CDR** | | **BNT** | | **Duration** | |
| --- | --- | --- | --- | --- | --- | --- | --- | --- | --- | --- | --- | --- |
|  | r | *p* | r | *p* | r | *p* | r | *p* | r | *p* | r | *p* |
| **VB1** | -0.269 | **0.010** | -0.009 | 0.929 | -0.052 | 0.616 | -0.140 | 0.469 | 0.014 | 0.915 | -0.073 | 0.441 |
| **VB2** | -0.219 | **0.036** | 0.029 | 0.778 | 0.000 | 0.997 | 0.111 | 0.566 | -0.210 | 0.111 | 0.013 | 0.891 |
| **VB3** | -0.050 | 0.636 | 0.038 | 0.716 | 0.020 | 0.847 | 0.092 | 0.634 | -0.222 | 0.092 | 0.004 | 0.967 |
| **VB5** | -0.114 | 0.279 | 0.158 | 0.127 | 0.227 | **0.028** | -0.265 | 0.165 | 0.210 | 0.110 | -0.115 | 0.225 |
| **VB6** | -0.027 | 0.796 | -0.096 | 0.354 | -0.046 | 0.660 | -0.443 | **0.016** | 0.105 | 0.427 | -0.187 | **0.048** |
| **VB7** | 0.070 | 0.509 | 0.111 | 0.285 | 0.153 | 0.140 | -0.123 | 0.526 | 0.301 | **0.021** | 0.087 | 0.360 |
| **VB9** | -0.344 | **0.001** | 0.002 | 0.986 | -0.098 | 0.347 | -0.029 | 0.881 | 0.022 | 0.869 | -0.028 | 0.770 |
| **VC** | -0.145 | 0.169 | 0.086 | 0.408 | 0.062 | 0.555 | -0.002 | 0.990 | 0.066 | 0.622 | 0.065 | 0.494 |
| **VB12** | -0.319 | **0.003** | 0.078 | 0.471 | 0.148 | 0.175 | -0.149 | 0.440 | 0.171 | 0.207 | -0.064 | 0.525 |
| **FA** | -0.440 | **<0.001** | <0.001 | 1.000 | -0.087 | 0.423 | -0.268 | 0.159 | -0.106 | 0.438 | -0.023 | 0.816 |
