# Supplementary material for: Lower water-soluble vitamins and higher homocysteine are associated with neurodegenerative diseases
Source: Sci Rep. 2025 May 29;15:18866. doi: 10.1038/s41598-025-03859-y (PMC12122920; doi:10.1038/s41598-025-03859-y)
Supplement: Supplementary file 2 — Supplementary Material 2 [file 41598_2025_3859_MOESM2_ESM.docx]

Supplementary materials

The list of analytes, corresponding compounds name, Cas No., and MRM transitions

| Analytes | Compounds name | CAS No. | Q1 mass (Da) | Q3 mass (Da) |
| --- | --- | --- | --- | --- |
| Vitamin B1 | Thiamine | 70-16-6 | 265.30 | 144.00 |
| Vitamin B2 | Riboflavine | 83-88-5 | 377.20 | 172.00 |
| Vitamin B3 | Nicotinamide | 98-92-0 | 123.30 | 78.10 |
| Vitamin B5 | Pantothenic acid | 79-83-4 | 220.00 | 72.00 |
| Vitamin B6 | 4-Pyridoxic acid | 82-82-6 | 184.06 | 147.94 |
| 5mTHF | 5-methyltetrahydrofolate | 31690-09-2 | 460.29 | 180.12 |
| Vitamin C | Ascorbic acid | 50-81-7 | 177.00 | 95.00 |
